# Consumers’ recommendations for improving access to healthcare services to adolescents with disabilities in Ghana

**DOI:** 10.1186/s13104-025-07267-3

**Published:** 2025-05-13

**Authors:** Ebenezer Mensah Gyimah, Ebenezer Dassah, Maxwell Peprah Opoku, Wisdom Kwadwo Mprah, William Nketsia, Afua Ntoaduro, Philip Atta Mensah, Cecilia Opoku, Clement Osei Tutu, Issaka Zakia, Richard Adade, Paul Okyere

**Affiliations:** 1 Department of Clinical Sciences, Auckland University of Technology, Auckland, New Zealand; 2 Department of Health Promotion and Disability Studies, Kwame Nkrumah University of Science and Technology, Kumasi, Ghana; 3 Department of Global and International Health, Kwame Nkrumah University of Science and Technology, Kumasi, Ghana; 4 Department of Special Education, United Arab Emirates University, Al-Ain, United Arab Emirates; 5 School of Education, Western Sydney University, Sydney, Australia; 6 Department of Interdisciplinary Studies, Akenten Appiah Menka University of Skills Training and Entrepreneurial Development, Kumasi, Ghana; 7 Bonn Centre for Dependency and Slavery Studies, University of Bonn, Bonn, Germany

**Keywords:** Consumers, Adolescents with disabilities, Families, Healthcare access, NHIS, Ghana’s Act 715

## Abstract

**Introduction:**

In Ghana, Adolescents with mobility and visual impairments and their families, encounter healthcare disparities, including socio-economic, physical, structural and attitudinal challenges, compared to the general population. Nonetheless, there is limited motivation to understand how to improve healthcare access for these marginalized consumers (i.e., adolescents with disabilities and their families). Consequently, the study explored marginalized consumers’ recommendations for improving access to healthcare services, described in Ghana’s Act 715. Informed by the qualitative descriptive design, forty-five (45) participants were purposively recruited and interviewed, adopting a semi-structured interview guide. Data gathered were subjected to content analysis and interpreted via lenses of the theory of dimensions of healthcare access.

**Results:**

The study’s findings were layered within areas of improving policy-practice interphase. Consumers called on policy makers to restructure the NHIS to cover indirect costs of accessing healthcare services for adolescents with disabilities. In respect of improving practice, it was also suggested that training and education on disability phenomenon must be extended to all cadres of healthcare workers including administrative staff, who provided services to this marginalized social unit. Participants further recommended enhancing the availability of healthcare workers and services as well as ensuring respect and protection of human rights within healthcare facilities.

## Introduction

Ratification of the United Nations Convention on the Rights of Persons with Disabilities (UNCRPD) mandates states to make provisions for the highest possible standard of healthcare (i.e., Article 25 of the UNCRPD) for Persons with Disabilities (PWDs) within local legislative instruments [1] Ghana has ratified the UNCRPD [2] and consequently passed the Person with Disability Act 2006 (Act 715), which makes provision for accessible healthcare services for all PWDs. Notwithstanding these legislative commitments, PWDs and their families often face significant barriers to accessing healthcare services [3] including economic, physical, structural and attitudinal barriers [3–6].

Adolescents with disabilities (i.e., self-reported visual and mobility impaired aged 13 to 18 years) commonly exhibit health behaviors that include limitations in activities of daily living such as independently standing, walking, climbing stairs, bending, reaching and grasping without much assistance [7]. In order that the nuanced healthcare needs of this group of marginalized consumers are effectively addressed by healthcare policies, it is essential that health policymakers acknowledge their access challenges. In particular, delays in accessing healthcare could exacerbate disabling conditions through degeneration of mild impairments into chronic and secondary conditions, and thus result in poorer health outcomes for PWDs [4, 8].

Persistent challenges faced by PWDs in accessing healthcare services suggest that existing instruments (i.e., UNCRPD and Ghana Persons with Disability Act, Act 715) have failed to improve healthcare outcomes for this marginalized group of consumers. Unfortunately, studies drawing on adolescents and their families with respect to health seeking behaviour are very few. The current study engaged adolescent with disabilities and their families on ways to enhance healthcare access to the former.

Penchansky and Thomas’ Healthcare Access Framework [9] was adopted as a theoretical lens to explore the complexity of healthcare affordability, timeliness, availability, geographic accessibility, and acceptability [10, 11] within marginalized populations. This framework analyzes how demographics, systemic and need factors shape access, making it suitable for investigating healthcare access for adolescents with disabilities and their families in Ghana.

## Methods

### Study design

The qualitative descriptive design was adopted to explore healthcare access among marginalized groups [12], designing an interview guide after the literature review based on the study’s objectives [3, 4, 6, 13–22].

### Study setting

The research took place in the Mampong Municipality in the Ashanti Region of Ghana hosting 24 healthcare facilities [23] with a disability population of 743 persons, including 154 children. The setting was selected based on the first author’s familiarity with the socio-cultural context, which enhanced data collection [24].

### Study population/inclusion criteria

Inclusion criteria for adolescents with disabilities were: aged between 13 and 18 years; having mobility or visual impairment(s), (i.e., prevalent impairments at study setting according to the records of Ghana Federation of Disability Organizations); able to communicate in Twi (the common language used at the study area) and/or English languages, and; willingness to participate. Families of adolescents with disabilities were also selected based on the following inclusion criteria: aged above 18 years; providing caregiving to an adolescent with disability spending a minimum of 20 h per week [25]; able to communicate in Twi and/or English languages, and; willingness to participate.

### Sample size, sampling and data collection

Using principle of “information power,” 45 participants (25 adolescents with disabilities and 20 unrelated families raising adolescents with disabilities) were purposively recruited based on their knowledge and experience. Data were collected through 45–60-minute interviews conducted predominantly in Twi language from March 10 to 20, 2023, focusing on consumers’ experiences in accessing healthcare under Ghana’s Disability Act 715.

### Data analysis

Data were analyzed through content analysis [26], by transcribing audio recordings, familiarizing with the texts, extracting key phrases, clustering similar themes, and interpreting the data. Participant validation was conducted to enhance credibility.

### Ethical consideration

Ethical approval was obtained from the Kwame Nkrumah University of Science and Technology’s Committee for Human Research, Publication and Ethics (CHRPE/AP/155/23), with consent secured from participants and Municipal offices of the Ghana Federation of Disability Organizations and Department of Social Welfare. All procedures complied with the Helsinki Declaration.

### Trustworthiness

Trustworthiness was ensured through member checking, reflexivity, and participant validation [27, 28].

## Results

### Healthcare services should be more affordable

Responses from participants showed the importance of making healthcare services more affordable, upholding the National Health Insurance Scheme (NHIS) as a critical social support system that facilitates access to healthcare. They noted that with NHIS, marginalized social units such as adolescents with disabilities can access almost all primary healthcare services without any charge. However, adolescents with disabilities discussed that the “*NHIS requires restructuring so that access to non-primary healthcare services could be more affordable*.” They further espoused that restructuring the NHIS could potentially prevent the worsening of their health conditions through expediting access to services beyond primary healthcare, such as physiotherapy for the mobility impaired.

Participants also highlighted the significance of including indirect healthcare costs in the expenditure borne by the NHIS. Specifically, adolescents with disabilities mentioned that the NHIS in its current structure did not cover the costs of acquiring assistive technologies such as reading and walking aids, prescribed by healthcare professionals. Most families also echoed similar opinions suggesting that restructuring the NHIS would facilitate access to affordable healthcare services for adolescents with disabilities. They discussed that a restructured NHIS should cater for all indirect expenses including costs on consumables (such as assistive devices) and services not presently covered under the NHIS. Families reiterated that “*NHIS should be extended to cover costs of transporting adolescents with disabilities and their family-caregivers to medical facilities.”*

### Healthcare services and workers should be available

A remarkable strategy for enhancing access to healthcare services suggested by participants was to increase the availability of healthcare services and workers. Adolescents with disabilities, particularly, remarked that access to healthcare services would be enhanced through the establishment of more rehabilitation centres within local health facilities. They further noted that employment of more allied healthcare professionals including mobility trainers, physiotherapists and optometrists among others, would ensure timely access to quality healthcare. An adolescent living with a disability highlighted:

The costs of long travelling distances to the Municipal capital and sometimes regional capital to seek eye specialists can be avoided by establishing an eye clinic with a resident specialist at our local health centre (Adolescent with disability 02, Female).

Families echoed similar sentiments emphasizing the need for the provision of rehabilitation services in local health facilities to enhance equitable access to healthcare. They further called for a revamp of existing rehabilitation centres with modern technology and equipment required for the provision of holistic healthcare services.

Participants also indicated that the built environment within health facilities should be re-engineered to accommodate adolescents with disabilities. Some participants also suggested that a mobile team of rehabilitation experts could periodically provide outreach services on specific days across communities. A grandmother remarked:

With their current limited numbers, rehab experts can provide outreach every market day at zonal council capitals where rural folks can access their services without much struggle (Family 10, Female).

### Encourage respect and protection of human rights at healthcare facilities

Participants with disabilities identified respect and protection of human rights as a key recommendation. They particularly expressed the need for healthcare workers to protect the dignity of PWDs. For instance, they indicated a need for health providers to designate special locations/spaces in health facilities for discussing confidential issues such as sexual and reproductive health services. Some families echoed similar views asserting the need for healthcare providers to protect the rights of adolescents with disabilities to privacy and dignified services:

Since sensitive information about our impairment is always in the public domain, I recommend that no third parties are allowed in consulting rooms within clinics. A lot more disabled people will access healthcare when our privacy and confidentiality is prioritized.(Adolescent with disability 16, Male).

Can you imagine, whilst sitting in a queue at the OPD, a nurse instructed me to pull down the dark (glasses) my daughter was wearing whilst she examined her watery and swollen eye? Considering how stigmatized disability is, I could not comply. We were so embarrassed. Therefore, I suggest that such conversations must be held alone in consulting rooms (Family 13, Female).

### Expansion of education and training on disability phenomenon

Participants noted that access to healthcare services could be enhanced through integrating disability issues in the curricula of health training institutions as well as, establishing continuous professional development on disability for healthcare workers. Most participants expressed that continuous in-service training would enable healthcare workers, including non-clinical staff to be aware of disability issues and provide optimal services to all. They mentioned that because healthcare workers provide services to all persons, including PWDs, they should have knowledge of how to meet their unique needs.

You see you cannot give what you don’t have. Especially, the healthcare administrators appear to have no clue about dealing with disabled persons. I propose adding disability studies to health training programmes if we require them to treat us well. (Adolescent with disability 06, Female)

I suggest health authorities also train the administrators and accountants at healthcare centres on how to serve the disabled because they just don’t treat us right. Imagine my daughter with mobility difficulties being asked to join a queue to… (Family 04, Female).

## Discussion

The discussion integrates participants’ recommendations with dimensions of healthcare access theory espoused by Penchansky and Thomas, to propose a multifaceted approach to improving healthcare access for adolescents with disabilities and their families [9]. Generally, the study’s findings highlight critical areas for enhancing healthcare access for adolescents with disabilities, identifying affordability, availability, respect for human dignity and education as key pillars [10].

Affordability remains a central concern, with participants emphasizing the need for NHIS to undergo restructuring to include coverage of indirect healthcare costs. Such a reconfiguration would align with broader conceptualizations of healthcare affordability [3], recognizing that access extends beyond the point of service to encompass all related expenses that contribute to the overall cost of care. Addressing these costs can mitigate financial barriers preventing the worsening of health conditions, and underlining the interconnectedness of economic factors and health outcomes.

The necessity for greater availability of healthcare services and professionals [11], particularly in rehabilitation and specialized care for adolescents with disabilities was highlighted. Enhancing infrastructure and workforce capacity in these areas can directly address the specific needs of this marginalized group, reducing delays in care and improving healthcare outcomes. Improved physical and staffing infrastructure within healthcare facilities significantly enhances health outcomes through exacerbation of disabling conditions, emanating from degeneration of mild impairments into chronic and secondary conditions [4, 8].

Respect and protection of human rights within healthcare settings emerged as imperative, with participants advocating for privacy, confidentiality and respectful treatment. This aspect of healthcare access focusing on dimensions of acceptability and awareness is crucial for maintaining the dignity of marginalized consumers through fostering an environment where they feel safe and valued. Such an environment can significantly enhance the willingness of adolescents with disabilities to seek and engage in healthcare services [29].

Furthermore, the call for integrating education on disability issues into healthcare training programs and continuous professional development is prudent to bridge knowledge gaps and enhance the competencies of both clinical and non-clinical healthcare providers, ensuring that the healthcare system can better accommodate the diverse and unique needs of adolescents with disabilities effectively. This recommendation is rooted in the awareness dimension of healthcare access [10], highlighting the importance of improving communication and understanding between providers and patients.

## Conclusion

The study highlights affordability, availability, respect for human rights, and education as key pillars for improving healthcare access for adolescents with disabilities. Drawing from participants’ experiences and the healthcare access framework, these findings offer practical insights for policymakers, healthcare providers, and educators to dismantle barriers and foster equitable healthcare. The findings call for systemic changes to promote accessible, dignified, and just healthcare for this marginalized group, advancing both public health and social justice.

## Limitations

This research was limited by its qualitative methodologies. Employing quantitative approaches and involving disability experts could deepen understanding of access to healthcare services for marginalized populations. Specifically, future studies could consider policymakers’ perspectives to evaluate effectiveness of healthcare policies in enhancing healthcare access for this group.

## Data Availability

The datasets used and/or analysed during the current study are available from the corresponding author on reasonable request.
